# Predictors of confidence in anatomy knowledge for work as a junior doctor: a national survey of Australian medical students

**DOI:** 10.1186/s12909-018-1280-5

**Published:** 2018-07-31

**Authors:** John E. Farey, David T. Bui, David Townsend, Premala Sureshkumar, Sandra Carr, Chris Roberts

**Affiliations:** 1 Royal Prince Alfred Hospital, Missenden Road, Camperdown, NSW 2050 Australia; 2 Nepean Hospital, Kingswood, NSW 2747 Australia; 3 Hunter New England Area Health Service, Maitland, NSW 2320 Australia; 4 Northern Clinical School, The University of Sydney, Palmerston Rd, Hornsby, NSW 2077 Australia; 5 Faculty of Health and Medical Sciences, The University of Western Australia, Crawley, WA 6009 Australia

**Keywords:** Anatomy, Medical education, Dissection, Confidence, Gender, Integration, Assessment

## Abstract

**Background:**

Major changes to the teaching of anatomy associated with the integration of basic and clinical sciences in modern medical curricula have coincided with students reporting concern over achievement of learning outcomes in anatomy. Little guidance exists for medical educators designing anatomy courses that account for factors that positively influence medical student confidence in their own anatomy knowledge. We sought to determine what factors are associated with medical students’ self-reported confidence in their anatomy knowledge in preparation for clinical practice.

**Methods:**

Cross-sectional national survey of Australian medical students distributed using social media. We performed univariate and multivariable ordinal regression to determine the factors in anatomy learning and teaching that influence medical student self-reported confidence to have sufficient anatomy knowledge by the time of graduation, for practice as a junior doctor.

**Results:**

Of 1309 surveyed, 1101 (84%) responded, representing 6.5% of the Australian medical student population. Mean age was 23.9 years (SD 4.8 years), a majority were female (644, 58.5%), and students in all years of both undergraduate (52%, 575) and graduate entry courses (48%, 529) were represented. Items associated with increased self-reported confidence in anatomy knowledge included adequate assessment of anatomy (Odds Ratio 2.17 [95% CI 1.69–2.81]), integration of anatomy with other basic sciences (OR 1.97 [1.52–2.56]) and clinical teaching (OR 1.90 [1.46–2.48]), male gender (OR 1.89 [1.48–2.42]), anatomy education prior to medical school (OR 1.46 [1.14–1.87]) and exposure to dissection (OR 1.39 [1.08–1.78]). Medical students in their clinical years reported lower confidence in their anatomy knowledge (OR 0.6 [0.47–0.77], *p* < 0.0001). Age and career intention were not significant predictors of confidence.

**Conclusions:**

Medical educators can enhance student confidence in their own anatomy knowledge by developing curricula that vertically integrating anatomy learning and teaching, integrate anatomy teaching with other basic sciences, and providing consistent assessment through both the pre-clinical and clinical stages of medical training. Anatomy education should also incorporate dissection as a teaching method, and students could benefit from completion of anatomy education prior to medical school. Consideration should also be given to further investigate the confidence of female students in their anatomy knowledge.

## Background

A sound knowledge of anatomy is important for work as a junior doctor, forming a cornerstone of diagnostic, procedural and clinical practice [1]. Anatomy is recognised as an essential core component of medical curricula [2], however medical students report feeling inadequately prepared in anatomy, ranking it as the discipline where they felt least prepared for postgraduate training [3]. At the same time, many clinicians and anatomists internationally have expressed concern over a perceived deficiency in the anatomy knowledge of recent medical graduates [4–9].

Various explanations for this phenomenon have been offered by researchers, including generational conflict, teaching of anatomy by non-medically qualified teachers, decreased use of dissection as a teaching tool and the use of integrated curricula such as problem based learning. So far, there remains insufficient evidence to support any one explanation [10]. Bergman et al., proposed four promising research areas in anatomy education, which would positively impact medical student knowledge of anatomy. These included: the vertical integration of anatomy education throughout the medical program; consistent assessment of anatomy knowledge to drive learning behaviour; and the teaching of anatomy in context to aid knowledge retention and diagnostic reasoning. They also stressed the importance of assessing student’s perception of anatomy teaching and the factors students feel influence their own learning [10]. Students in PBL courses may be more likely to perceive deficiencies in their anatomy knowledge, leading to anxiety in their ability to achieve the intended learning outcomes [11, 13].

An opportunity to address the gap in the literature around students preparedness for practice in the context of anatomy knowledge arose in a secondary analysis of national survey of Australian medical students. This larger study explored the demographic characteristics, teaching factors and curriculum design factors associated with medical students’ self-reported confidence that they would have sufficient anatomy knowledge by the time they graduated, for practice as a junior doctor. Findings of this current study were intended to provide guidance for medical educators designing anatomy curricula, so they may incorporate the factors that positively influence medical student confidence in their own anatomy knowledge.

The Australian medical education environment consists of a hybrid of both European-style undergraduate and North American-style postgraduate medical programs, with medical programs increasingly shifting towards the latter over the past two decades [14]. The rise of four-year graduate programs has led to both an increase in the number of students with prior basic science and anatomy knowledge entering medical school, and a decrease in the amount of time available to teach anatomy. At the same time, the gender composition of medical students in Australia has changed, with female medical graduates outnumbering males [15, 16]. Students intending a career in an anatomy-heavy discipline such as surgery or radiology are more likely to rate themselves confident in anatomy knowledge [17, 18], but less is known regarding other demographic factors that may influence confidence, such as gender and age.

In this paper, we asked the research question “What are the important factors in anatomy learning and teaching that are associated with increased self-reported confidence in anatomy knowledge to be prepared for practice as an intern.”

## Methods

### Questionnaire development

A self-administered 19-item questionnaire was developed in accordance with best practice guidelines [19]. Drawing on key themes from the literature, we conducted four focus groups with students from five different universities. The findings were synthesised into a draft questionnaire, and subjected to validation by six experts in medical education research or anatomy teaching who were identified during a preliminary literature review. Demographic questions were developed based on previously validated government health workforce planning instruments, including a list of the 27 distinct specialist training pathways available in Australia [3, 15]. The draft questionnaire was pilot tested by a random group of 10 medical students for validity, assessment of item range and variance, as well as content and clarity of wording, prior to the release of the final version of the survey.

### Setting & participants

All medical students enrolled at any Australian medical school in 2015 were eligible to participate in the survey. The survey was distributed via the national Australian Medical Students’ Association (AMSA) and locally based medical student societies through use of the social network application, Facebook. Recruitment was open for 10 weeks between September and November 2015 and was promoted through a series of Facebook posts, which featured a promotional message and graphic, with a link to the survey. This message was shared 3 times per week over the recruitment period. Participants were able to separately enter their details into a draw for two small financial incentives of $250 Australian dollars following completion of the survey. This amount is consistent with similar medical student surveys [20] and was provided by the authors.

### Data collection

We captured three types of data: demographic characteristics, teaching factors and curriculum design factors. Demographic characteristics included age, gender, length of medical course, undergraduate or graduate entry status, current year of medical course, university, as well as specialty career intention. Teaching factors included use of dissection, and formal anatomy education prior to commencement of medical school. Curriculum factors included three questions on frequency of assessment, level of integration of anatomy with basic sciences teaching as well as vertical integration of anatomy into clinical teaching. These factors were measured using a five-point Likert scale, consisting of ‘far too little’, ‘too little’, ‘about right’, ‘too much’ or ‘far too much’. The outcome variable of interest, whether students were confident that they would have sufficient anatomy knowledge by the time they graduated, for practice as a junior doctor, was measured on an ordered six-point Likert scale with three levels of agreement and three level of disagreement. Reported levels of confidence were subsequently collapsed into three categories of ‘unconfident’, ‘undecided’ and ‘confident’ to aid univariate and multivariate analysis. We used the online platform Survey Gizmo (Boulder, CO, USA) to host the survey instrument.

### Data analysis

Data were analysed using SAS (version 9.1.4) statistical software. Univariate and multivariate ordinal regression models using cumulative logits were used to assess the association between the perceived level of confidence and variables of interest using Odds Ratios. The Chi-squared score test for the proportional odds assumption was used to assess whether the main model assumption was violated. Descriptive statistics are presented as counts and percentages.

### Ethic approval and consent for participation

The study was approved by the Human Research Ethics Committee of the University of Western Australia (RA/4/1/7753). All participants provided written consent, and were assured of the voluntary nature of participation before providing consent.

## Results

### Demographics

Of 1309 respondents consenting to participate, 1101 respondents completed the survey and 208 provided partial responses which were excluded from the final analyses, giving a completion rate 84%. Table 1 lists the characteristics and career intentions for all 1101 complete responses. The mean age of respondents was 23.9 years (SD 4.8 years), 58.5% were female, and the five most common future career intentions were surgery, internal medicine, emergency medicine, general practice, and paediatrics. Slightly greater than half of the respondents were undergraduate students (52%, 572/1101).

**Table 1:** Characteristics and career intentions of 1101 medical students questioned about their anatomy teaching exposures and perceived confidence to apply that knowledge after graduation. Values are numbers (percentages) of responses

Characteristics	No. (%)
Age (years)	
< 20	106 (9.6)
20–24	653 (59.4)
25–29	245 (22.3)
30–34	56 (5.1)
35–39	23 (2.1)
40+	17 (1.5)
Gender	
Male	457 (41.5)
Female	644 (58.5)
Length of Medical Course (years)	
4	529 (48.0)
5	318 (28.9)
6	254 (23.1)
Undergraduate or Graduate Entry	
Undergraduate	572 (52.0)
Graduate	529 (48.0)
Stage of Medical Course	
1st Year	245 (22.3)
2nd Year	268 (24.3)
3rd Year	204 (18.5)
4th Year	202 (18.3)
5th Year	113 (10.3)
6th Year	69 (6.3)
Pre-Clinical or Clinical Years	
Pre-Clinical	638 (57.9)
Clinical	463 (42.1)
Formal Anatomy Unit of Study Prior to Medical School?	
Yes	424 (38.5)
No	677 (61.5)
Exposure to Dissection	
Yes	705 (64.0)
No	396 (36.0)
Career Intention^a^	
Surgery	204 (18.5)
Internal Medicine	181 (16.4)
Emergency Medicine	146 (13.3)
General Practice	141 (12.8)
Paediatrics	110 (10.0)
Obstetrics and Gynaecology	72 (6.5)
Anaesthesia	62 (5.6)
Other	185 (16.8)
Amount of time dedicated to Anatomy Assessment?	
Too little	496 (45.7)
About right or too much	590 (54.3)
Amount of time dedicated to integrating Anatomy with Clinical teaching?	
Too little	612 (57.5)
About right or too much	453 (42.5)
Amount of time dedicated to integrating Anatomy with other Basic Sciences?	
Too little	526 (48.7)
About right or too much	555 (51.3)
Confidence for anatomy knowledge in internship	
Confident	291
Undecided	581
Unconfident	228

^a^ Only career intentions with greater than 5% of respondents have been included

### Factors that predict confidence in anatomy knowledge after graduation

Univariate analysis demonstrated six elements to be significantly positively associated with medical student’s having increased confidence that they would have sufficient anatomy knowledge by the time they graduated, for practice as a junior doctor. In descending order, these were a) vertical integration of anatomy instruction with clinical teaching (OR 3.14 [2.36–3.81]), b) increased frequency of assessment of anatomy knowledge (OR 3.00 [2.36–3.81]), c) integration of anatomy teaching with other basic sciences teaching (OR 2.85 [2.25–3.62]), d) male gender (OR 2.10 [1.66–2.65]), e) formal anatomy instruction prior to medical school (OR 1.67 [1.32–2.11]) and f) exposure to dissection (OR 1.42 [1.12–1.80]) (*p* < 0.0001) (Table 2). Medical students in their final clinical years of study were 48% less likely to be confident that they would have sufficient anatomy knowledge by the time they graduated, when compared to medical students in the earlier pre-clinical years of study (OR 0.52 [0.41–0.66]).

**Table 2:** Predictors for Confidence in Anatomy Knowledge for Internship

Medical student characteristics (No.)	Unadjusted Odds Ratio (95% CI)	P	Adjusted Odds Ratio (95% CI)	P
Age	0.99 (0.97–1.02)	0.57	–	–
Gender				
Female	1.00	< 0.0001	1.00	< 0.0001
Male	2.10 (1.66–2.65)		1.89 (1.48–2.42)	
Formal anatomy course prior to commencement of medical school				
No	1.00	< 0.0001	1.00	< 0.0001
Yes	1.67 (1.32–2.11)		1.46 (1.14–1.87)	
Stage within medical course				
Pre-clinical years	1.00	< 0.0001	1.00	< 0.0001
Clinical years	0.52 (0.41–0.66)		0.60 (0.47–0.77)	
Career intention				
Surgery	1.00	0.09	–	0.69
Anaesthesia	0.66 (0.38–1.13)			
Emergency Medicine	0.77 (0.51–1.15)			
General Practice	0.71 (0.47–1.07)			
Internal Medicine	0.85 (0.58–1.24)			
Obstetrics & Gynaecology	0.43 (0.26–0.71)			
Paediatrics	0.67 (0.43–1.05)			
All other specialties	0.82 (0.56–1.20)			
Exposure to dissection				
Not Exposed	1.00	< 0.0001	1.39 (1.08–1.78)	0.01
Exposed	1.42 (1.12–1.80)			
Frequency of assessment				
Too little	1.00	< 0.0001	1.00	< 0.0001
About right or too much	3.00 (2.36–3.81)		2.17 (1.69–2.81)	
Integration with clinical teaching				
Too little	1.00	< 0.0001	1.00	< 0.0001
About right or too much	3.14 (2.46–4.01)		1.90 (1.46–2.48)	
Integration with basic sciences				
Too little	1.00	< 0.0001	1.00	< 0.0001
About right or too much	2.85 (2.25–3.62)		1.97 (1.52–2.56)	

In the multivariable analysis, all six elements significantly predicted increased confidence in medical students (Table 2). An association between career intention and confidence in anatomy knowledge was demonstrated in the univariate analysis, but this was not found to be statistically significant in the multivariate analysis.

## Discussion

Our data demonstrates there are several significant factors which are associated with increased medical student confidence in anatomy knowledge in the context of being prepared for practice as an intern. These in decreasing order were; 1) vertical integration of anatomy instruction with clinical teaching, 2) increased frequency of assessment of anatomy knowledge, 3) integration of anatomy teaching with other basic sciences, 4) male gender, 5) formal anatomy instruction prior to medical school, 6) exposure to dissection, and 7) being in the pre-clinical years of the medical degree.

Our data suggest that the four modifiable factors could be incorporated into medical curricula design in order to provide an integrated approach to achieving meaningful learning outcome in anatomy. These factors are vertical integration of anatomy instruction with clinical teaching, increased frequency of assessment of anatomy knowledge, and integration of anatomy teaching with other basic sciences. These three factors partly resonate with Bergman et al.’s narrative review on why students don’t know enough about anatomy [10]. The need for vertical integration is also supported by the observation that clincial students were less confident in their anatomy than junior students. That exposure to dissection might impact preparedness for practice as an intern is a new finding.

### Implications

We now explore more fully the implication of both the modifiable factors in terms of curriculum design in order of their contribution to student confidence in anatomy knowledge.

#### Vertical Integration

Our results also support the vertical integration of anatomy teaching throughout the entirety of pre-clinical and clinical teaching. The majority of survey respondents (57.5%) indicated there was insufficient integration of anatomy teaching in clinical years, with the students in their clinical years reporting the lowest confidence in their anatomy knowledge for work as a junior doctor. While clinically relevant applications of basic science have been heavily integrated into the pre-clinical curriculum, there has been less progress in integrating anatomy teaching into the clinical curriculum [6]. Medical schools could offer electives in anatomy for interested students during the clinical years. They could also shift the teaching of complex anatomy to the later years of the medical course, when students can learn clinically relevant anatomy in context, which has been demonstrated to aid retention of basic sciences and clinical knowledge as well as improve diagnostic reasoning [10].

#### Assessment

A significant proportion of survey respondents (54.7%) felt that there was too little assessment of anatomy in their medical course. Some studies have found that low relative weighting of anatomy in assessments leads to other areas of study being prioritised. Anecdotal accounts from Australia medical students suggest some are entirely forgoing anatomy study due to low assessment weighting [21]. A study evaluating the impact of assessment weighting in one UK medical school demonstrated an association between increasing assessment weighting in anatomy as part of an overall grade and reported higher motivation towards learning the subject [22]. Where health sciences graduates have been surveyed, they have favoured regular practical assessment using cadavers and imaging over written and oral formats [23]. Although the quality of anatomy assessment remains an underdeveloped area in the literature and needs further study [10], our results demonstrate that Australian medical students feel that quantity of anatomy assessment is insufficient in their medical courses, consequently reducing student confidence in their own anatomy knowledge.

#### An integrated learning experience

The strong association between the integration of integrated anatomy instruction and confidence in anatomy knowledge in our study suggest that Australian medical students endorse the integration of anatomy and basic sciences in modern medical curricula. This finding is likely to be contentious among clinical anatomists and surgeons who have argued that we should return to the era of anatomy as a standalone discipline within the medical curricula [5, 6]. There is little evidence in support of this argument [11, 12] and any perceived deficiencies in anatomy knowledge are likely to be related to factors other than curriculum integration [4, 24, 25].

#### Demographic factors

The results of our multivariate analysis suggest that career intention is not predictive of perceived confidence in anatomy knowledge for work as a junior doctor. Prior evidence has found that students intending a surgical career are more likely to rate their anatomy education as inadequate [17, 18], but has not examined the relationship between career intention and confidence. We regard this as a particularly important negative finding, as it indicates that students intending a career in an anatomy-laden discipline such as surgery are no more likely to report a ‘confident’ level of anatomy knowledge compared to students wishing to pursue a career in general practice, for example. The implication of this is that any expressed student concern regarding adequacy of anatomy teaching is likely to be shared among a broader group of students, not just those agitating for increased anatomy relevant to their future surgical career.

The influence of gender on confidence in anatomy knowledge is another area that has had little exploration in the literature [17]. Males in our cohort were nearly twice as likely to be confident in their anatomy knowledge for internship than females, and the number of males listing surgery as their preferred career was also close to double that of their female colleagues. The general effect of gender on confidence is well established in the literature; while males and females consistently demonstrate parity in standardised clinical examinations, females are more likely to rate their levels of confidence as lower [26]. What is less clear is whether the apparent selection bias of males for surgical careers negatively impacts the confidence of female students, potentially dampening their enthusiasm for anatomy during medical school.

#### Prior exposure to anatomy

Little has been written on the value of pre-requisites in anatomy prior to the commencement of medical school as part of the application process into medical school. Our findings show the positive impact that anatomy education prior to medical school has on confidence in anatomy knowledge. In Australia, only one of the twelve medical schools offering a four-year graduate-entry program requires satisfactory completion of specific units in anatomy prior to application [27]. The decreased emphasis on anatomy in modern medical curricula disadvantages students without prior anatomy education, putting the onus on medical faculties to ensure these students achieve an adequate level of anatomy knowledge during their training. Elective anatomy units may be one way of addressing this potential shortfall.

#### Dissection

The decline of dissection and cadaver-based teaching in anatomy education has been perhaps one of the most controversial aspects of changes to medical school curricula in recent years. Despite the historical prominence of dissection in medical education, the rationale for its use as a teaching method has stemmed from tradition, rather than a demonstrated impact on learning outcomes [28, 29]. A recent survey of Australian medical faculties revealed that dissection is still available in at least 12 of the 20 medical schools with a graduating cohort in 2015 [30], coinciding with a trend towards increasing use of alternative teaching methods over the past two decades [7, 21].

Our study showed that exposure to dissection had a positive impact on medical student confidence in anatomy knowledge, and other student evaluations of dissection as a teaching method have previously shown strong support for the method in attaining key learning outcomes [7]. Students have also shown a preference for dissection over modern methods such as computer-assisted learning [31].

### Limitations

The use of social media for survey recruitment introduces a degree of selection bias. We are unable to determine what percentage of Australian medical students were exposed to the survey link, however we do know that social media use is almost universal among Australian medical students [20], and the demographic proportions in our sample are largely consistent with the national medical student population [16]. While all respondents to the survey were required to complete a declaration stating that they were currently enrolled as a medical student at an Australian medical school, there was in effect no barrier to members of the public completing the survey if provided the survey link. This remains a disadvantage of social media as a research recruitment method.

We acknowledge that self-reported confidence in anatomy knowledge does not equate to competence. This has previously been demonstrated in studies of medical student and junior doctor performance in practical and simulation-based assessments [32]. We also acknowledge that medical students are unable to judge what level of anatomy knowledge they will require for safe clinical practice. A British study of 140 graduating medical students found that 68.3% were concerned about their level of anatomy knowledge at time of graduation, but following a year of clinical practice, 77% reported having received enough anatomy education at medical school to practice competently [13]. Self-reported confidence in anatomy knowledge, as a domain specific construct, represents a measure of academic self-efficacy rather than a measure of general confidence as a personality trait [33] and as discussed by Bergman & Van der Vleuten [10], we contend that identifying the factors that influence medical student academic self-efficacy in anatomy is important for effective curricula design.

Our survey does not address quality of assessment, and we acknowledge that learning is influenced by many factors, including the learning environment, quality of instruction and resources. Finally, there is the potential for recall bias as our survey instrument relied on students self-reporting teaching method exposures, curriculum and demographic factors.

## Conclusion

Medical educators can enhance student confidence in their own anatomy knowledge by developing curricula that integrate anatomy teaching with other basic sciences, while ensuring that anatomy teaching and consistent, regular assessment is vertically integrated through both the pre-clinical and clinical stages of medical training. Anatomy education should also incorporate dissection as a teaching method, and at least some anatomy education could be encouraged for all students prior to commencing graduate-entry courses where appropriate. Consideration should also be given to investigating the self reported lack of confidence of female students in their anatomy knowledge. In particular to determine whether this has any influence on the noted lower interest of female students in pursuing surgical careers.

Future research could investigate whether the assessment and curriculum factors that have been identified in this study to positively influence student confidence also have a corresponding positive impact on anatomy related academic performance and clinical practice.
